# Conformational diversity and protein–protein interfaces in drug repurposing in Ras signaling pathway

**DOI:** 10.1038/s41598-023-50913-8

**Published:** 2024-01-12

**Authors:** Ahenk Zeynep Sayin, Zeynep Abali, Simge Senyuz, Fatma Cankara, Attila Gursoy, Ozlem Keskin

**Affiliations:** 1 Department of Chemical and Biological Engineering, College of Engineering, Koc University, Rumeli Feneri Yolu Sariyer, 34450 Istanbul, Turkey; 2 Graduate School of Science and Engineering, Computational Sciences and Engineering, Koc University, 34450 Istanbul, Turkey; 3 Department of Computer Engineering, Koc University, 34450 Istanbul, Turkey

**Keywords:** Computational biology and bioinformatics, Target identification

## Abstract

We focus on drug repurposing in the Ras signaling pathway, considering structural similarities of protein–protein interfaces. The interfaces formed by physically interacting proteins are found from PDB if available and via PRISM (PRotein Interaction by Structural Matching) otherwise. The structural coverage of these interactions has been increased from 21 to 92% using PRISM. Multiple conformations of each protein are used to include protein dynamics and diversity. Next, we find FDA-approved drugs bound to structurally similar protein–protein interfaces. The results suggest that HIV protease inhibitors tipranavir, indinavir, and saquinavir may bind to EGFR and ERBB3/HER3 interface. Tipranavir and indinavir may also bind to EGFR and ERBB2/HER2 interface. Additionally, a drug used in Alzheimer's disease can bind to RAF1 and BRAF interface. Hence, we propose a methodology to find drugs to be potentially used for cancer using a dataset of structurally similar protein–protein interface clusters rather than pockets in a systematic way.

## Introduction

Drug repurposing or drug repositioning, using a drug for an indication other than its original purpose, is an attractive option compared to the long and costly process of developing a new drug^1,2^. The drug to be repurposed has already been studied for its safety and has extensive data on its pharmacokinetics. As a result, many stages of drug development can be omitted^3^. Some examples of successfully repurposed drugs are thalidomide and sildenafil. Thalidomide, an antiemetic drug for pregnant women that was subsequently proven to have teratogenic effects, has been repurposed to be used in leprosy and sildenafil, a drug originally developed for angina, has been used in erectile dysfunction^4^. Current drug repurposing cases typically follow a disease-centric approach, but when disease-focused repurposing reaches its limits, target-centric and drug-centric repurposing relying on structural data will be crucial^5^. Docking and virtual screening are some of the most common methods in computational drug repurposing for preliminary studies^6^. Some of the structure-based virtual screening web servers for drug repurposing are ACID (using inverse docking approach^7^), DRDOCK (combining docking and molecular dynamic simulations for a target protein^8^), and MTiOpenScreen (using docking or blind docking^9^).

Cell signaling is the transmission of an external signal to activate certain mechanisms in the cell^10^. Ras/Raf/MEK/ERK signaling pathway plays a role in the transduction of a signal received from an extracellular receptor to the cell nucleus to regulate biological functions, including cell proliferation, differentiation, apoptosis, and stress response^11–14^. Dysregulation of this pathway is associated with diseases such as inflammation, developmental disorders, neurodegenerative disorders^11,15–17^ and is observed in approximately one-third of all human cancers^18^. Consequently, various drugs targeting this pathway have been developed. Vemurafenib, dabrafenib, and trametinib are some examples of MAPK inhibitors used in cancer therapy^19^. Proteins in this pathway interact with other proteins and these interactions take place through protein–protein interfaces^20^. Hence, protein–protein interfaces are critical targets for drugs to regulate abnormal protein–protein interactions (PPIs) in this pathway^21,22^. Disruption of a PPI by targeting the interface with a drug may interrupt the transduction of a signal that promotes tumorigenesis, thereby being beneficial in cancer therapy^23^. From our previous studies, we know different proteins can form similar protein–protein interface architectures^24–26^. Using similar interfaces, Engin et al.^27^ proposed that drugs binding to an interface might also bind to another interface with a similar structure. Their case study showed that the drugs binding to the interface between CDK6 and CDKN2D also bind to the interface between CDK4 and CDKN2D, which has a similar interface, with comparable binding energies.

**Figure 1:**
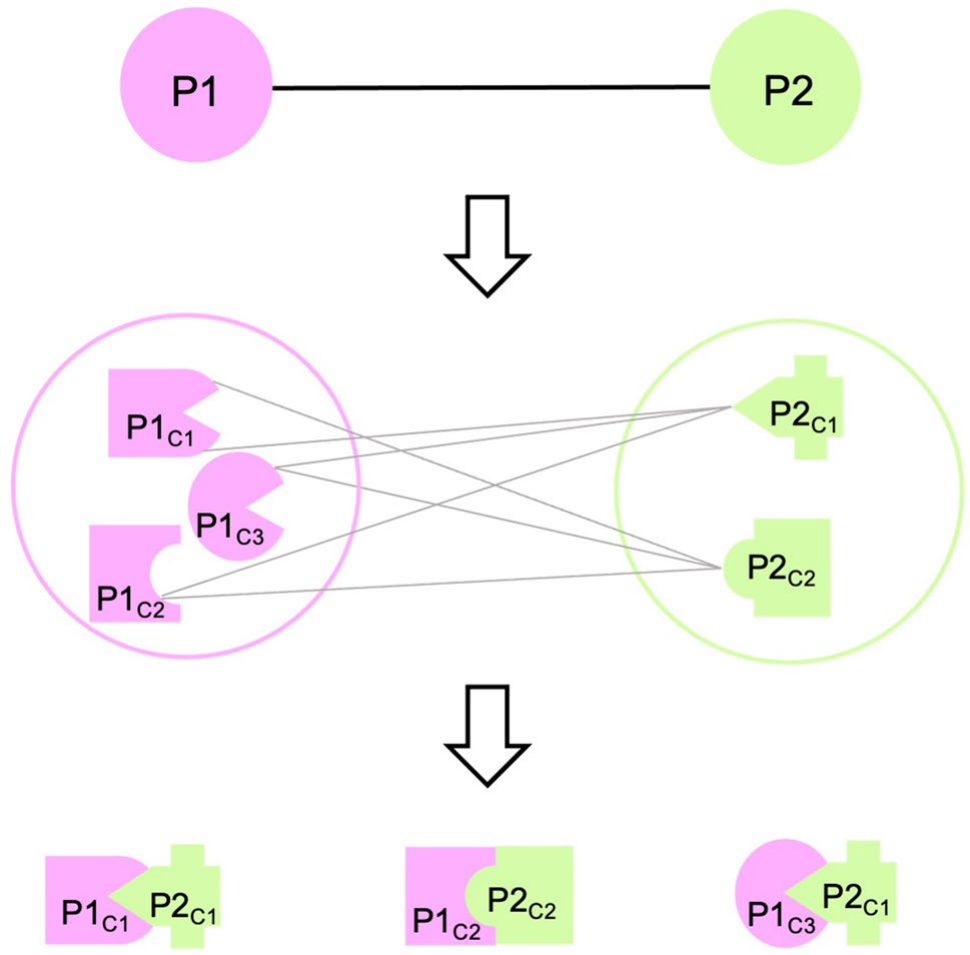
Protein–protein interactions with multiple conformations. Interacting proteins, Protein 1 (P1) and Protein 2 (P2) have three and two conformations, respectively. Considering the multiple conformations of each protein, there are six possible interactions in theory (represented with grey edges) but in reality, only some of these interactions can be found (three of them in this case).

Here, our aim is to use a non-redundant protein–protein interface dataset that is clustered based on structural similarity for drug repurposing. We preferred studying protein–protein interfaces rather than the binding pockets because target proteins may sometimes lack binding pockets, limiting their druggability, such as in the case of RAS protein family^28–30^. Moreover, molecular glue is a new concept that may be used to make the targets druggable, which were once considered as undruggable and the protein–protein interfaces are perfect for this approach^31^. In this study, we focused on protein–protein interfaces of Ras/Raf/MEK/ERK signaling pathway. We studied interfaces that are available in Protein Data Bank (PDB)^32^ and used PRISM web server^33,34^, a prediction tool for protein–protein interactions at the structural level, to predict the interfaces between any two physically interacting proteins of Ras/Raf/MEK/ERK signaling pathway when there is no experimental data. Proteins are dynamic and the conformational space is diverse. The availability of different conformations is crucial to finding the right one that fits the drug molecule and PDB is getting richer with many conformations for a single protein. Here, we used an ensemble of conformations rather than taking a single structure in the predictions (Fig. 1). Considering alternative conformations of each protein, the number of successfully predicted interactions increases^35^. We extracted drugs already bound to the interfaces as candidates of drug repurposing for target proteins with structurally similar interfaces. Finally, we performed docking to propose drugs to be repurposed and found literature evidence showing that the algorithm we used here can be promising in suggesting new uses for already known drugs.

## Results

The Ras/Raf/MEK/ERK signaling pathway is reconstructed by 16 proteins in the KEGG database^36–38^ under the EGF–EGFR–RAS–ERK signaling pathway and their top 10 interactors according to STRING database^39^. All available structures of these 26 proteins in PDB^32^ are grouped based on sequence and structural similarity. The representatives of the alternative conformation groups of these proteins can be found in Supplementary Table S1. These conformations either correspond to the alternative conformations of the same region or may correspond to different parts of a protein. The pathway proteins have 4.56 ± 5.20 conformations on average. The structures of GAB2 in PDB have less than 30 amino acids and are eliminated in the grouping process. Its AlphaFold^40,41^ model is used in the following steps.

**Figure 2:**
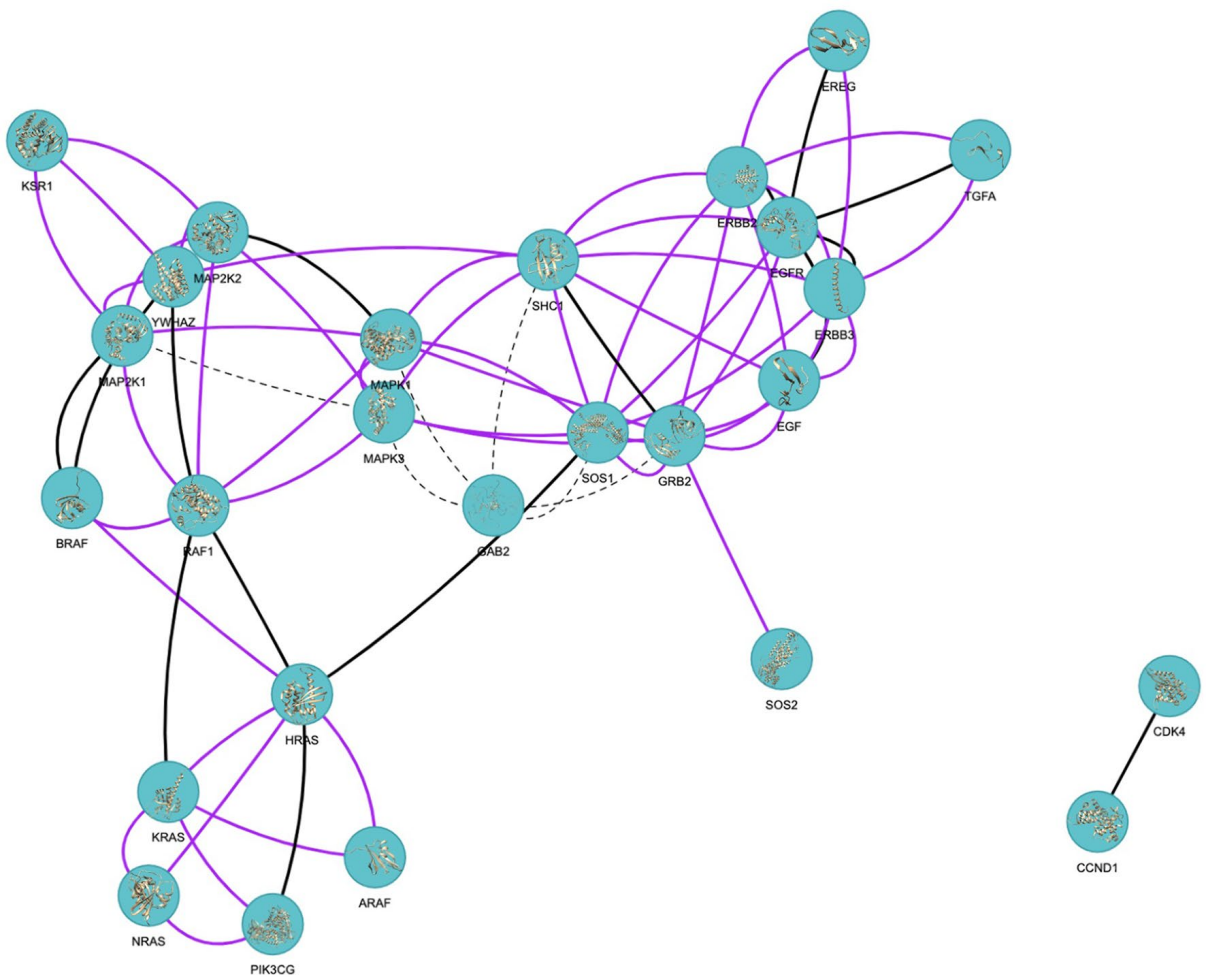
Protein–protein interaction network of Ras/Raf/MEK/ERK signaling pathway. Nodes represent proteins and proteins connected by edges represent the interaction between those proteins. If the edge is black, the complex of interacting proteins is available in PDB. If the edge is purple, the complex is not available in PDB but is predicted by PRISM. If the edge is a dashed line, the complex is neither available in PDB nor predicted by PRISM^42^.

The network of Ras/Raf/MEK/ERK signaling pathway consists of 26 proteins and 72 interactions. When the alternative conformations of each protein are considered, there are 2564 possible interactions in theory, resulting from 72 interactions between 26 proteins. Figure 1 shows this concept: one interaction results in six interactions in theory between alternative conformations in Fig. 1. Interacting protein pairs considering alternative conformations are submitted to the PRISM web server^33,34^. PRISM simulations predicted 3309 complexes for 66 of 72 interactions reported in STRING. The number of the predicted complexes is more than the number of possible interactions because PRISM may predict more than one protein–protein complex structure for a pair of proteins. These interactions can be seen in Fig. 2. With the PRISM predictions, the structural coverage of protein–protein complexes formed by physically interacting proteins of this pathway has been increased from 15 to 66 out of 72 interactions found on STRING^39^ with the highest confidence score (≥ 0.900). These results correspond to 999 of all 2564 possible interactions among alternative conformations and through 630 unique protein interface templates (Supplementary Text S1). The results involve some complexes for the same protein structures with different binding energy predictions. All the predictions are used in the next steps to avoid missing any new targets.

Additionally, there are 994 PDB structures, 521 of which have more than one chain, involving at least one of the 16 proteins in the EGF–EGFR–RAS–ERK signaling pathway. In total, 1296 protein interfaces were formed in these PDB entries. These interfaces are combined with the interfaces predicted by PRISM.

**Figure 3:**
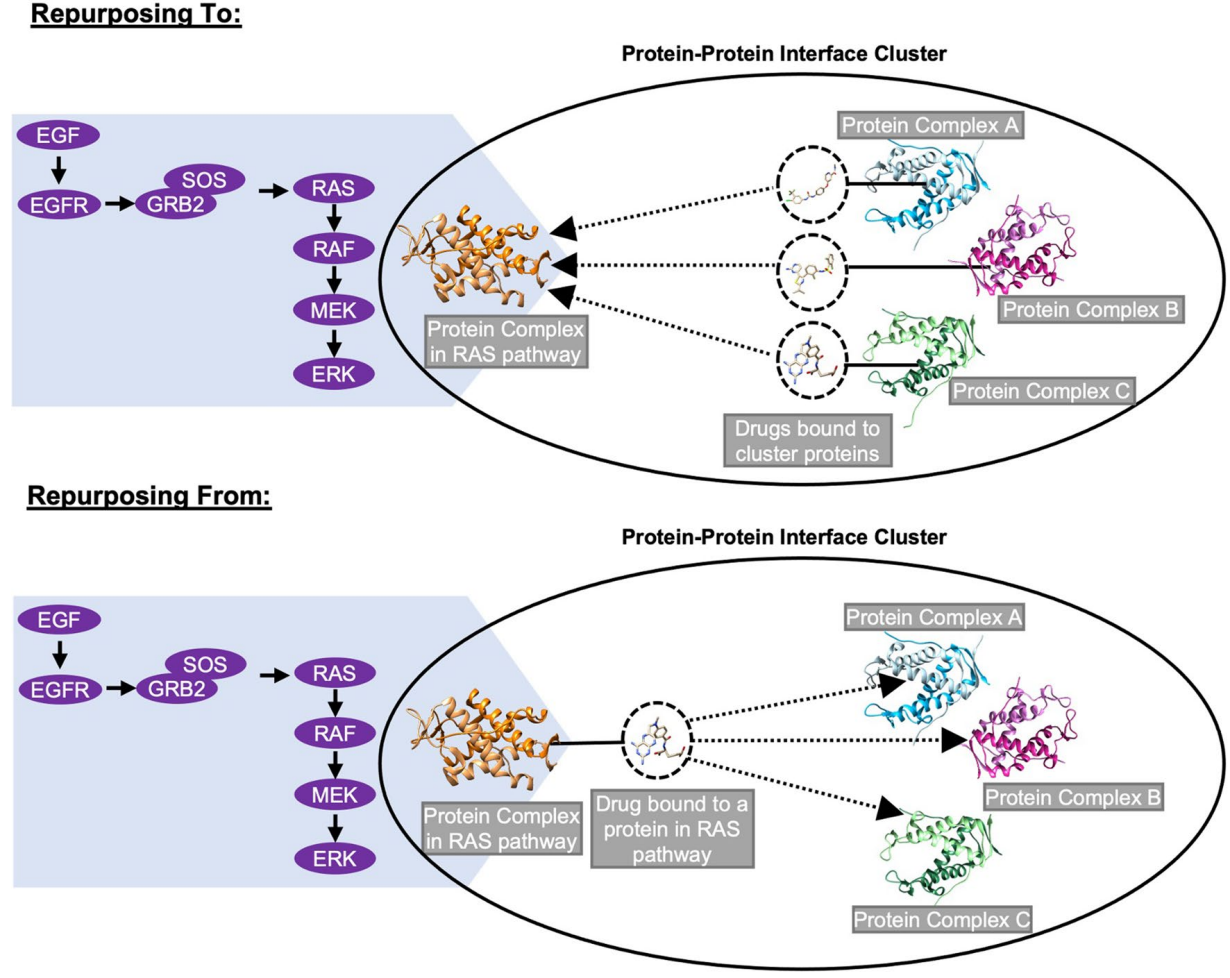
Identification of new drug–target pairs. A solid line represents a drug bound to an interface. Identified new drug–target pairs are represented with a dotted line with an arrow.

A structurally non-redundant dataset of protein–protein interface clusters (Supplementary Dataset S3)^43^ is used to find possible new drug–target pairs. A schematic representation of two scenarios is shown in Fig. 3. The first one is “Repurposing To”, where a drug bound to one of the interfaces in a protein–protein interface cluster may bind to an interface in the same cluster that belongs to the Ras/Raf/MEK/ERK pathway. In the second scenario of “Repurposing From”, a drug bound to a protein interface in Ras/Raf/MEK/ERK pathway may also bind to another protein interface that is in the same cluster, and the protein is not in the Ras/Raf/MEK/ERK pathway.

We filtered the clusters that contain all the template interfaces of the PRISM predictions and experimental PDB structures for the “Repurposing To” strategy. With the approach of “Repurposing To”, there are 441 and 71 possible new drug–target pairs from PRISM results and PDB entries of pathway proteins, respectively (Supplementary Table S2).

Considering unique protein interfaces of PRISM predictions, there are five different FDA-approved drugs bound to six different protein interfaces. In contrast, there are eight protein interfaces with three different FDA-approved drugs among the interfaces related to EGF–EGFR–RAS–ERK signaling pathway in PDB entries. The mentioned interfaces and drugs can be seen in Supplementary Table S3. These protein interfaces are used for the “Repurposing From” strategy (see “Methods” for details). With the approach explained as “Repurposing From”, we have 72 possible new drug–target pairs from PRISM predictions and 120 from protein interfaces in pathway PDB entries (Supplementary Table S4).

We further performed docking for these drug–target pairs and the results were analyzed according to the binding free energy (Supplementary Table S5). A previous study proposed an average binding energy of − 7.75 ± 0.06 kcal/mol^44^. Accordingly, new targets are presented in Table 1. These proteins contain both intracellular domains or extracellular domains and a result between intra- and extracellular regions is not biologically meaningful. Therefore, we eliminated such cases.

**Table 1:** Proposed drug repurposing candidates.

Chain 1^a^	Chain 2^a^	Drug name	Ligand ID	Original interfaces with the drug^b^	ΔG (kcal/mol)
ERBB3 (4LEO_C)	**EGFR** **(4UIP_A)**	Tipranavir	TPV	HIV-1 Protease (1D4S_A_B)	− 7.334
RAF1 (6XGU_B)	**BRAF** **(6Q0K_A)**	Granisetron	CWB	Mutant Binding Protein (5HTBP-AchBP) (2YME_A_B)	− 7.580
ERBB3 (4LEO_C)	**EGFR** **(4UIP_A)**	Indinavir	MK1	HIV-II Protease (1HSH_A_B)	− 7.532
RAF1 (6XGU_B)	**BRAF** **(6Q0K_A)**	Galantamine	GNT	Ach-binding Protein (2PH9_C_D)	− 7.382
**ERBB3** **(4LEO_C)**	EGFR (4UIP_A)	Saquinavir	ROC	HIV-1 Protease (1HXB_A_B)	− 7.379
**ERBB2** **(3N85_A)**	EGFR (1YY9_A)	Tipranavir	TPV	HIV-1 Protease (1D4S_A_B)	− 7.351
**ERBB2** **(3N85_A)**	EGFR (1YY9_A)	Indinavir	MK1	HIV-II Protease (1HSH_A_B)	− 7.302

^a^The chains that the drug is bound are in bold.

^b^Only one of the interfaces that the drug is originally bound to is provided in the table. All interfaces are presented in Supplementary Table S6.

To compare the binding energies of randomly selected drugs to these interfaces, a control set of 35 drugs is docked to the interfaces presented in Table 1. The average binding energy of these drugs to the protein–protein interfaces is − 5.57 kcal/mol, whereas the median is − 5.52 kcal/mol. When the distribution of the binding energies is assessed (Supplementary Fig. S1), it is seen that there is a drastic decrease in the number of docking scores below − 7.33 kcal/mol, which is consistent with our cut-off value for the proposed drug repurposing candidates.

**Figure 4:**
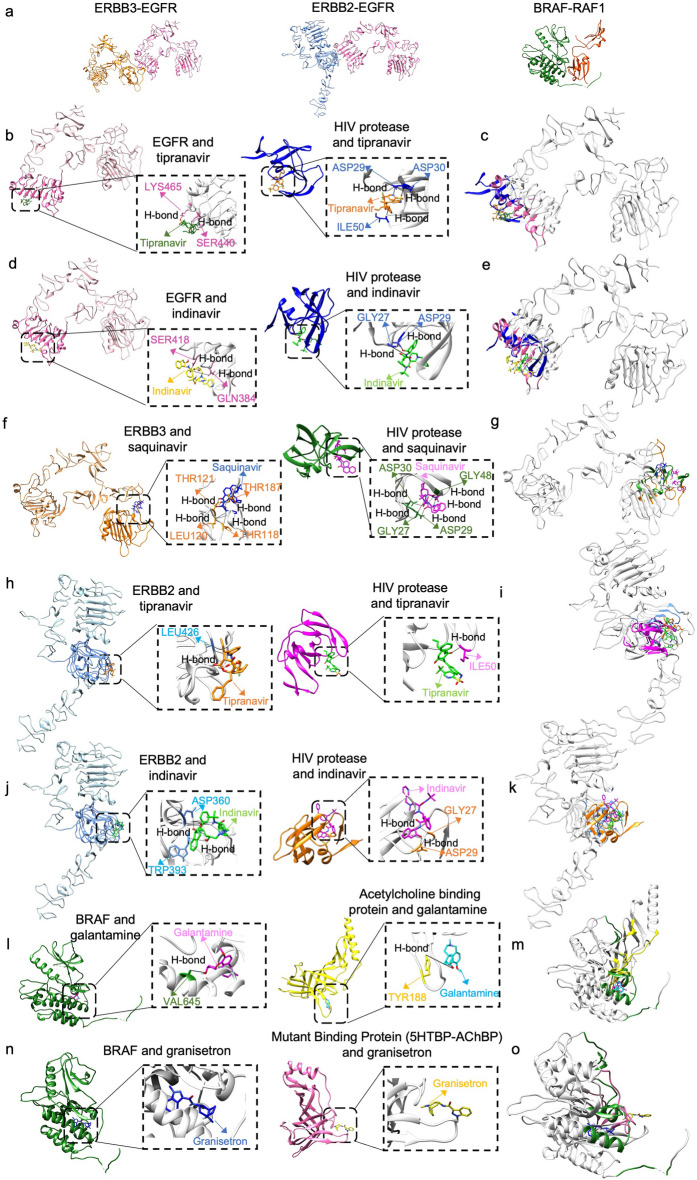
ERBB3–EGFR, ERBB2–EGFR, RAF1–BRAF interface results. (**a**) ERBB3–EGFR, ERBB2–EGFR and BRAF–RAF1 complexes. (**b**) Tipranavir with EGFR (4UIP_A), represented in pink with domain III in darker shade^32,47^, and its original target HIV protease (1D4S_A). (**c**) Structural alignment of EGFR and HIV protease interfaces with tipranavir^45^. (**d**) Indinavir with EGFR (4UIP_A), represented in pink with domain III in darker shade^32,47^, and its original target HIV protease (1HSH_A). (**e**) Structural alignment of EGFR and HIV protease interfaces with indinavir^45^. (**f**) Saquinavir with ERBB3 (4LEO_C), represented in orange with domain I in darker shade^32,47^, and its original target HIV protease (1HXB_A). (**g**) Structural alignment of ERBB3 and HIV protease interfaces with saquinavir^45^. (**h**) Tipranavir with ERBB2 (3N85_A), represented in blue with domain III in darker shade^32,47^, and its original target HIV protease (1D4S_B). (**i**) Structural alignment of ERBB2 and HIV protease interfaces with tipranavir^45^. (**j**) Indinavir with ERBB2 (3N85_A), represented in blue with domain III in darker shade^32,47^, and its original target HIV protease (1HSH_A). (**k**) Structural alignment of ERBB2 and HIV protease interfaces with indinavir^45^. (**l**) Galantamine with BRAF (6Q0K_A) and its original target acetylcholine binding protein (2PH9_C). (**m**) Structural alignment of BRAF and acetylcholine binding protein with galantamine^45^. (**n**) Granisetron with BRAF (6Q0K_A) and its original target mutant binding protein (2YME_A). (**o**) Structural alignment of BRAF and mutant binding protein with granisetron^45^ (Molecular graphics performed with UCSF Chimera, developed by the Resource for Biocomputing, Visualization, and Informatics at the University of California, San Francisco, supported by NIH P41-GM103311.0^48^. Ligand–protein interactions and structural alignment are performed with LigPlot^46^ and MultiProt^45^, respectively).

Table 1 presents the protein–protein interfaces proposed for drug repurposing (columns 1 and 2) and the protein complexes (column 5) with a drug bound to their interfaces that have structural similarity to the proposed interfaces. These protein–protein interfaces, the drug binding protein chain of the protein–protein interface, the protein that the drug is originally bound to and the structural alignment^45^ of their interfaces can be seen in Fig. 4. Tipranavir and indinavir form two hydrogen bonds with EGFR, while saquinavir forms four hydrogen bonds with ERBB3 at the ERBB3–EGFR interface^46^. Tipranavir and indinavir at the interface of ERBB2–EGFR have one and two hydrogen bonds with ERBB2, respectively^46^. The hydrogen bond between indinavir and ASP29 of HIV protease is maintained between indinavir and ASP360 of ERBB2. Moreover, the hydrogen bond between the galantamine and its original interface is also present between galantamine and BRAF-RAF1 interface^46^. Lastly, granisetron interacts with BRAF through hydrophobic contacts^46^ as it does with mutant binding protein (5HTBP-AChBP), which is the protein that it is originally bound to in PDB. Furthermore, the interfaces of the original targets and the proposed new targets of the drugs are structurally aligned according to MultiProt^45^ results. The RMSD of the matched interface residues are 1.72 Å, 1.85 Å, 1.88 Å, 1.88 Å, 1.74 Å, 1.53 Å and 1.53 Å for the structural alignments in Fig. 4c, Fig. 4,e,g,i,k,m,o, respectively. In Fig. 4, it can be seen that the drugs are binding to the same region of the aligned structures.

**Figure 5:**
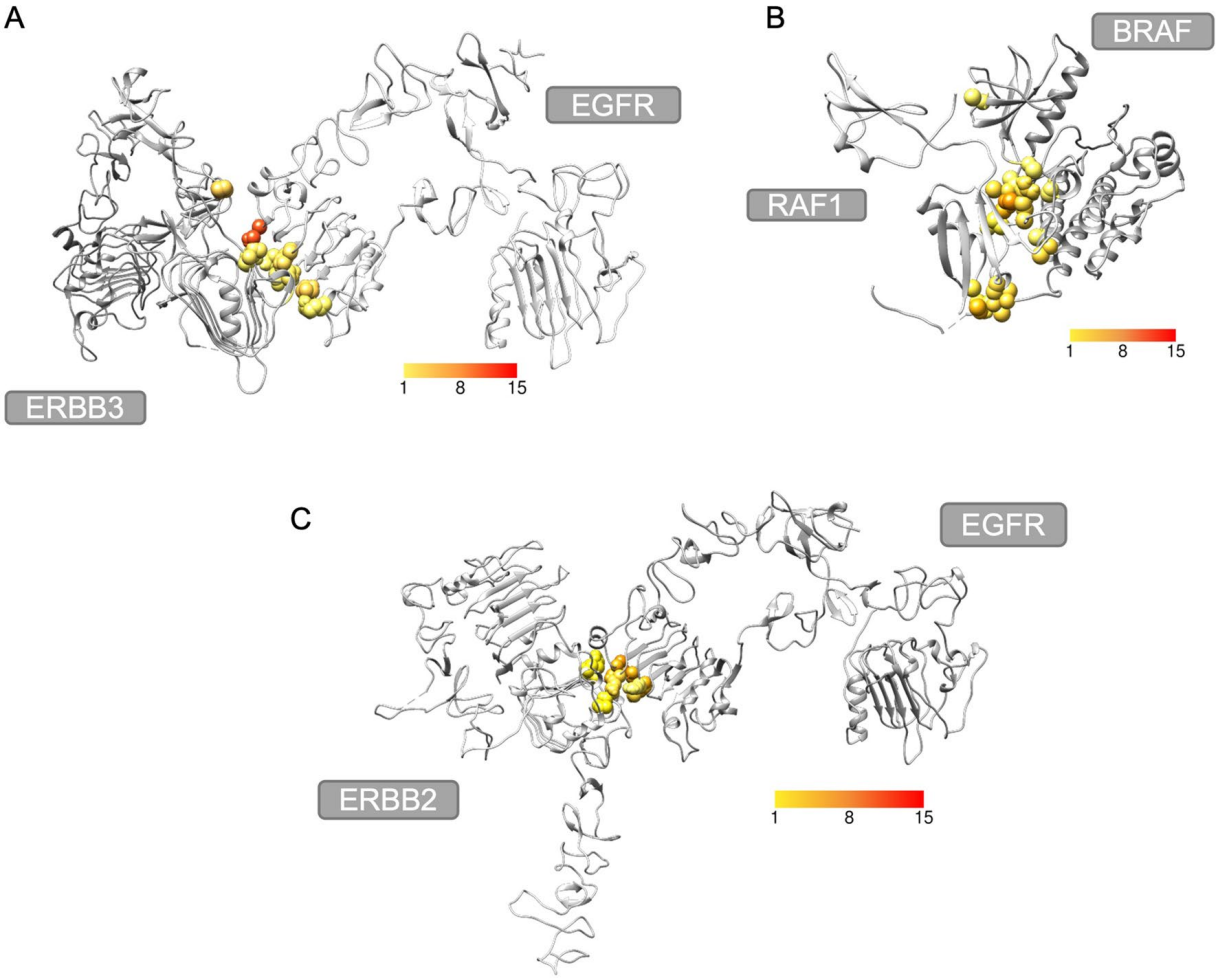
Cancer mutations at the interface of the protein–protein complexes (**a**) ERBB3–EGFR complex with cancer mutation residues colored according to frequency. (**b**) BRAF–RAF1 complex with cancer mutation residues colored according to frequency. (**c**) ERBB2–EGFR complex with cancer mutation residues colored according to frequency.

Since mutations at the interface may alter the protein–protein interactions and the interaction with ligands, residues where cancer mutations are observed are extracted from the COSMIC database^49^. Then, they are mapped to the interface residues and the effect of the mutations on the protein function is predicted by SIFT^50^. The cancer mutations located at the interfaces (of the protein–protein complexes in Table 1) can be seen in Fig. 5. At the interface of the ERBB3–EGFR complex, ERBB3 has two mutations predicted as deleterious to the protein function out of six mutations located at the interface, but only one of the deleterious mutations (Q138L) is at the ligand contacting residues (i.e., with a distance of less than 5 Å) of the proposed drug repurposing candidates. On the other hand, EGFR has eight residues at the interface where cancer mutations are observed, but none of them are predicted to be deleterious by SIFT. There are four residues of RAF1 and thirteen residues of BRAF related to cancer mutations at the interface of BRAF–RAF1 complex. The mutations of RAF1 are predicted as functionally neutral, but six of BRAF mutations are predicted to be deleterious by SIFT. However, these deleterious mutations are not at the contacting residues of galantamine or granisetron. Moreover, ERBB2 has one deleterious mutation (P416T or P416L) at the contacting residues of indinavir and tipranavir among the three mutations located at the interface of ERBB2–EGFR complex. Lastly, one of the eight mutations located at the interface residues of EGFR is predicted to be deleterious (N444I) in addition to being one of the contacting residues of indinavir and tipranavir. Mapped mutations for all the complexes used in this study are presented in Supplementary Table S7 and Supplementary Table S8. The frequency and tissue information of the mutations from COSMIC database^49^ that are located at the interface of the protein–protein complexes in Table 1 and their predicted SIFT score can be found in Supplementary Table S9.

**Figure 6:**
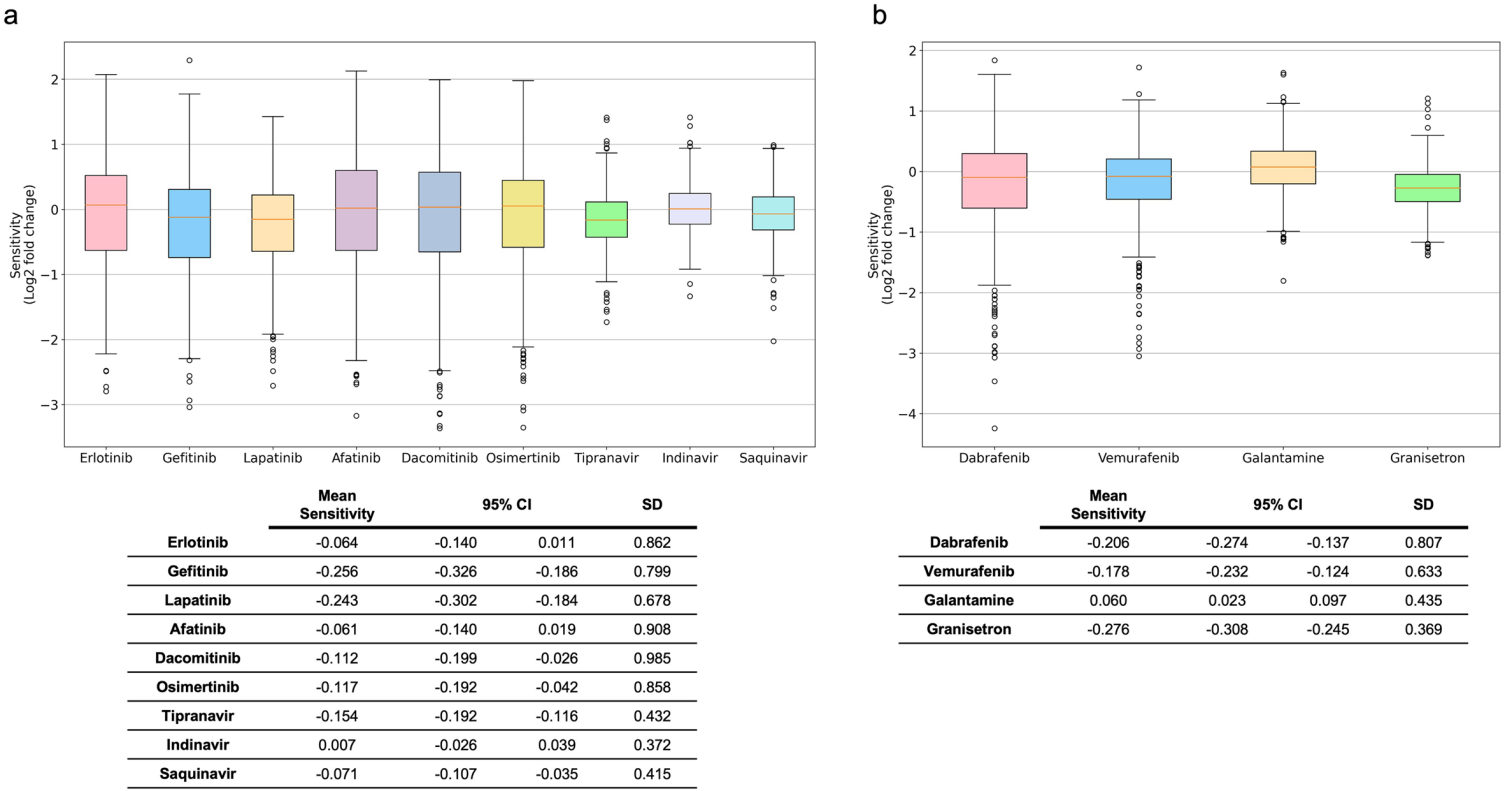
Sensitivity of cancer cells to cancer drugs and proposed drug repurposing candidates. (**a**) Sensitivity of cancer cells to EGFR/ERBB2 inhibitors erlotinib, gefitinib, lapatinib, afatinib, dacomitinib, and osimertinib in comparison to drug repurposing candidates tipranavir, indinavir, and saquinavir with their mean sensitivity, 95% confidence interval (CI) and standard deviation (SD). (**b**) Sensitivity of cancer cells to BRAF/RAF1 inhibitors dabrafenib and vemurafenib compared with drug repurposing candidates galantamine and granisetron and their mean sensitivity, 95% CI and SD.

The sensitivities of cancer cells to our proposed drug repurposing candidates and to the drugs that are used in cancer treatment are extracted from DepMap^51^. Erlotinib, gefitinib, lapatinib, afatinib, dacomitinib, and osimertinib are EGFR inhibitors, whereas lapatinib and afatinib also target ERBB2^52^. Cancer cells exposed to our proposed drug repurposing candidates tipranavir and saquinavir binding to EGFR-ERBB3 and/or EGFR-ERBB2 interfaces had less viability than the control group. Moreover, cancer cells were more sensitive to tipranavir and saquinavir than to erlotinib and afatinib, which are cancer drugs (Fig. 6a). Furthermore, higher drug sensitivity is observed with tipranavir on cancer cells compared to dacomitinib and osimertinib. Vemurafenib is a BRAF inhibitor, whereas dabrafenib targets both BRAF and RAF1. Granisetron, which is suggested to be binding to BRAF-RAF1 interface, leads to higher sensitivity in cancer cells than cancer drugs vemurafenib and dabrafenib (Fig. 6b). When the cell lines used in this analysis are grouped by their primary diseases, the group with the highest number of cell lines is non-small cell lung cancer, followed by melanoma and diffuse glioma (Supplementary Fig. S2).

## Discussion

Drug repurposing may adopt a ligand-based approach or target-based approach. Here, we used a new concept, the structural similarities of the protein–protein interfaces to propose new targets for the FDA-approved drugs in Ras signaling pathway. This method required the 3-dimensional structures of the protein–protein complexes. However, only 21% of the physically interacting protein complexes on STRING^39^ with the highest confidence score were available in PDB for our network. Using PRISM, a template-based structural prediction tool, structural coverage of the network is increased to 92% for these protein–protein interactions on STRING^39^. Thus, we could use more protein–protein interfaces in our further steps, such as filtering the interfaces with FDA-approved drugs and identifying new drug–target pairs to search for drug repurposing candidates. Here, the conformational diversity of proteins is integrated by using multiple conformations of the pathway proteins. For instance, if just one structure (PDB ID:3KSY Chain ID:A) of SOS1 had been used, protein–protein complex structures for only 60% of the listed interactions would have been found, but the value is increased to 90% using multiple conformations. After the prediction of the complexes that are not available in the literature, new drug–target pairs are identified using a structurally clustered protein–protein interface dataset. Drugs that are suggested for repurposing are determined according to their binding free energy prediction via docking (Table 1) to similar interfaces. The protein chain having a favorable binding energy for target–ligand complex is stated as the new target.

Three of the results involve EGFR-ERBB3 protein interface formed by structures with PDB IDs of 4UIP and 4LEO with chain IDs of A and C, respectively. EGFR is a transmembrane protein of ErbB family of tyrosine kinase receptors^53^. EGFR, also known as HER1, involves extracellular region comprising four domains, transmembrane region and intracellular region with tyrosine kinase domain^54,55^. Domain III of the extracellular region plays a role in ligand binding^53^. ERBB3, also known as HER3, is also a member of ErbB family and consists of three regions, namely extracellular, transmembrane and intracellular regions. Its extracellular region also has four domains among which domains I and III are involved in binding of its natural ligand heregulin^56^. EGFR and ERBB3 can form heterodimers as well as homodimers resulting in activation of MAPK/ERK and PI3K/Akt signaling pathways that are responsible for cell migration and proliferation^57–59^. Previous studies showed that EGFR-ERBB3 heterodimer is involved in signaling which promotes metastasis in melanoma cells and activation of MAPK^60^. According to another study, upregulation, mutation or catalytic activation of ErbB family proteins are associated with breast, ovarian, colorectal, pancreatic, and lung cancer. Moreover, targeting a single protein in therapy might fail because of the crosstalk between ErbB family that activates downstream pathways. In that study, it is also reported that targeting the EGFR-ERBB3 interface for breast cancer is an improved strategy where malignancies exhibit resistance to treatment that targets a single protein^61^.

Structures with PDB IDs of 4LEO and 4UIP are extracellular domains of ERBB3 and EGFR, respectively. A previous study suggested that targeting the extracellular domain of EGFR is promising in colorectal cancer treatment where there is resistance to EGFR inhibitors cetuximab and panitumumab^62,63^. In our results, tipranavir and indinavir bind to EGFR with favorable binding energy at the interface formed between EGFR and ERBB3 (Fig. 4b,d). Both indinavir and tipranavir are drugs used in the treatment of HIV infection^52^. Tipranavir and indinavir were approved by FDA in 2005 and 1996, respectively^64,65^. They both bind to the active site of HIV protease enzyme to prevent hydrolysis of peptide bonds which is necessary for the life cycle of HIV^66^. According to docking results, tipranavir and indinavir are bound to domain III of EGFR extracellular domain. In another study, cetuximab which is an EGFR inhibitor is also bound to the domain III^67^ suggesting that these HIV protease inhibitors might be used in cancer treatment.

The other drug that binds to the same interface formed by these protein structures is saquinavir which is also an HIV protease inhibitor. Saquinavir binds to ERBB3 with a lower (better) energy. Saquinavir was approved in 1995, being the first HIV protease inhibitor approved by FDA^68^. Saquinavir is bound to the domain I of ERBB3 extracellular domain (Fig. 4f), which is one of the domains involved in ligand binding and inhibition may prevent activation of downstream signaling pathways that play a role in the growth of cancer cells.

Tipranavir and indinavir also bind to the interface formed between EGFR and ERBB2, with a lower binding energy to ERBB2 protein chain (Fig. 4h,j). The complex consists of chain A of structure with PDB ID 1YY9 and chain A of structure with PDB ID 3N85 representing EGFR extracellular domain and ERBB2 extracellular domain, respectively. ERBB2, also known as HER2, is another member of ErbB family. Thus, its extracellular domain consists of four subdomains where subdomains I and III are involved in ligand binding^69^ and subdomains II and IV play roles in homodimerization and heterodimerization^70^. EGFR-ERBB2 heterodimer activates MAPK pathway, preventing apoptosis^71^. Overexpression of ERBB2 is highly related to breast cancer and is observed in 20–30% of all breast cancers^72^. Upregulation of ERBB2 expression may promote cell proliferation and can further lead to tumorigenesis^73^. Amplification of ERBB2 also occurs in 10–30% of gastric cancers and has been associated with different types of cancer, such as ovary, colon, and bladder cancers^74–76^. Consequently, ERBB2 has become a therapeutic target of interest. Trastuzumab is a monoclonal antibody used in breast and gastric cancer and targets ERBB2^77,78^. There are also other therapeutic strategies that are developed for patients with trastuzumab resistance. Dual tyrosine kinase inhibitor lapatinib is one of them and targets both EGFR and ERBB2^79^. Moreover, recombinant humanized ERBB2 monoclonal antibody pertuzumab prevents dimerization of ERBB2 with EGFR and ERBB3 to prevent activation of downstream pathways, which is demonstrated to be inhibiting breast and prostate tumor growth^78,80^. Since tipranavir and indinavir bind to the domain III of ERBB2 extracellular domain and are at the EGFR-ERBB2 interface according to our results, they may disrupt the heterodimer and prevent the cell signaling. Therefore, these HIV protease inhibitors may be repurposed for tumor growth inhibition. There have been studies on repurposing of HIV protease inhibitor nelfinavir for cancer, suggesting its mechanism of action involves inhibition of MAPK signaling pathway^81,82^. Moreover, the phase II clinical trial of indinavir for non-HIV associated classic Kaposi’s Sarcoma reported positive outcome after receiving treatment for 61.5% of the patients^83^. Furthermore, a study demonstrated that tipranavir induced apoptosis of gastric cancer stem cells by targeting PRSS23-IL24 pathway^84^. Hence, the HIV protease inhibitors that we reported in our results may be repurposing candidates for cancer.

Regarding the toxicity of HIV protease inhibitors in combination with chemotherapies or radiotherapy, the clinical trials of nelfinavir for cancer therapy might give insight. In the phase I trial of nelfinavir in combination with chemoradiotherapy on unresectable stage IIIa/IIIb non-small cell lung cancer (NSCLC), Rengan et al. reported that nelfinavir administered 7 to 14 days before or at the same time with cisplatin, etoposide, and radiotherapy at a dose of 66.6 Gy resulted in no predetermined dose-limiting toxicity^81,85^. In the phase II trial conducted by 35 patients with IIIa/IIIb NSCLC by Rengan et al., no unexpected grade 3 or 4 toxicities were observed apart from those of standard chemoradiotherapy^81,86^. Moreover, Brunner et al. reported that nelfinavir with concurrent chemoradiotherapy did not exhibit any additional toxicity in the phase I clinical trial in inoperable locally advanced pancreatic cancer patients^81,87^.

The interface formed between RAF1 and BRAF also has drugs that have low binding energy. RAF1, also known as CRAF, and BRAF are both members of Raf kinase family along with ARAF. Their structure is comprised of three conserved regions (CR), namely, C1 with Ras-binding domain and cysteine-rich domain; CR2 with serine/threonine-rich region; and CR3 involving kinase domain^88^. Heterodimer of BRAF and RAF1 formation is induced by growth factor-stimulated RAS and activates MEK and ERK to promote cell proliferation, differentiation, survival, and migration^89,90^. BRAF-RAF1 heterodimer is the most active dimer compared to their homodimers in MEK1/2 activation^91,92^. BRAF mutation is observed in nearly 8% of all cancers and is mostly associated with melanoma^93^. Mutated RAF1 is less common in human cancers but mutation in RAF1 may lead to Noonan syndrome which is a disorder that includes short stature, facial dysmorphology, and congenital heart defects^94,95^. Also, it is reported that increased BRAF heterodimerization with RAF1 is associated with RAF1 mutations related to Noonan syndrome^96^. Since mutation in BRAF also promotes MAPK signaling pathway activation and tumorigenesis, it has been identified as a target in cancer therapy^91^.

According to our results, granisetron and galantamine bind to BRAF with favorable energy at the interface formed between BRAF (PDB ID:6Q0K Chain ID:A) and Ras binding domain and cysteine-rich domain of RAF1 (PDB ID:6XGU Chain ID:B) (Fig. 4l,n). Granisetron is a serotonin type 3 (5-HT3) receptor antagonist used as an antinauseant for cancer chemotherapy patients^97^. There are several studies where some other drugs binding to a serotonin receptor are proposed as anticancer agents. For example, tegaserod which is a serotonin receptor 4 (HTR4) agonist is reported to be inducing apoptosis in B16F10 murine melanoma cell line and some human melanoma cell lines by perturbing PI3K/Akt/mTOR pathway^98^. In another study, methiothepin which is a nonselective serotonin 5-HT receptor antagonist is reported to be increasing the efficacy of chemotherapy when used along with doxorubicin, against melanoma cells^99^. The same study shows that methiothepin also enhances the efficacy of BRAF inhibitor vemurafenib and MEK inhibitor trametinib, used against resistant BRAFV600E melanoma cells.

Galantamine is an acetylcholinesterase inhibitor used in the treatment of Alzheimer’s disease^100^. Abnormal expression of acetylcholinesterase is observed in several tumors, therefore, is associated with tumor development^101–106^. As a result, some acetylcholinesterase inhibitors may be considered as possible anti-cancer agents for the cancer types where increased activity of acetylcholinesterase is observed^107^. Inhibition of the MAPK pathway may be another mechanism when using acetylcholinesterase inhibitor galantamine as an anti-cancer agent.

Both granisetron and galantamine are bound to the kinase domains^108,109^ according to our results. BRAF inhibitors such as sorafenib also bind to the kinase domain of BRAF (PDB ID:1UWH) and if these drugs also act as BRAF inhibitors or disrupt the BRAF-RAF1 protein interface, they can be potential anti-cancer drugs. However, in some cases, a BRAF inhibitor such as vemurafenib, binding to BRAF leads to inhibition of BRAF but transactivation of RAF1 further leads to activation of MEK and ERK. To prevent paradoxical activation, a high level of RAF inhibitor that acts on both BRAF and RAF1 may be used^110^.

Additionally, somatic mutations in human cancers are mapped to interfaces of the 3-dimensional structures of the protein complexes used in this study via COSMIC database^49^ and SIFTS UniProt-PDB mappings^111^ on PDBe API^112^. COSMIC database^49^ provides manually curated mutation information of tumor samples including mutation types. Here, nonsense mutations that stop the translation prematurely and missense mutations that result in encoding of different amino acids at that location are mapped. For the ERBB3–EGFR complex, the interface mutation with the highest frequency for ERBB3 is observed in 0.006% of the samples and they are from endometrium, large intestine, and bile duct tumors. In contrast, the highest frequency for the interface mutations of EGFR is 0.007%, from the samples of large intestine and lung carcinoma (Supplementary Table S9). Recurrent ERBB3 mutations are observed in colon and gastric cancers and there are various studies on characterization of ERBB3 mutations in cancer^113,114^. However, the mapped ERBB3 mutations located at the interface of ERBB3–EGFR complex have not been characterized as oncogenic mutations in these studies^113,114^. Considering EGFR mutations at the interface, G465R and S492R are identified to be related to cetuximab resistance, while S492R does not affect panitumumab binding in colorectal cancer treatment^115,116^. Residue G465 of EGFR is one of the contacting residues of indinavir at ERBB3–EGFR interface in our study and might affect the binding. For the BRAF-RAF1 complex, the highest frequency of mapped mutations is 0.005% for RAF1 and they are from various tissues such as large intestine, brain, and endometrium. RAF1 mutations located at the interface of BRAF–RAF1 complex are checked in PanCancer Studies^117–126^ on cBioPortal^127^ and it is seen that their oncogenic effects are marked as unknown^127^. For BRAF, the highest frequency is 0.003% from mutations in the samples of ovary, lung, kidney, skin and lymphoid at the E586 position. The BRAF mutation E586K has been identified to be related to lung adenocarcinoma^128^ and kinase activity is increased in COS cells that exhibit this mutation^129^. Moreover, it is reported that HEK293 cells with BRAF E586K showed sensitivity to pan-RAF inhibitor (LY3009120) by inhibited phospho-MEK and -ERK activities^130^. The mutations E586K, H725Y, and H725Q are marked as likely to be oncogenic on cBioPortal^127^ but these mutations are not located at the contacting residues of granisetron or galantamine in our study. At the interface of ERBB2–EGFR complex, ERBB2 has mutations with the frequency of 0.002% observed in tissues like skin, ovary, and stomach. In contrast, the highest mutation frequency for EGFR is 0.007%, observed in large intestine and lung tissues. The ERBB2 mutations at the interface of ERBB2–EGFR complex are not mentioned as one of the activating/oncogenic mutations of ERBB2^131^ or available in cBioPortal^127^. On the other hand, EGFR mutations S464L, G465R, K467T, and S492R observed in cetuximab resistance in colorectal cancer treatment^115,116^ are located at the contacting residues of tipranavir and indinavir while I491M is also a contacting residue of indinavir in our study. The protein structures that we used in our studies do not exhibit these mutations and the mutations may change the protein–protein interactions and the interaction with the drug. However, not all of the people with cancer have these mutations considering the frequency of the mutations among the tumor samples, and not all of the mutations have a functional effect on the protein (Supplementary Table S9).

This study relying on structural similarities of protein–protein interfaces revealed that indinavir, tipranavir, and saquinavir originally used for HIV infection treatment may bind to EGFR-ERBB3 and/or EGFR-ERBB2 interfaces and can be repurposed for cancer treatment. Additionally, the Alzheimer’s disease drug galantamine and antiemetic drug granisetron may bind to BRAF-RAF1 interface and can be used as anti-cancer agents to prevent tumor growth. Even though these results present candidates for drug repurposing, they should be validated by experiments and clinical trials.

## Conclusions

Drug repurposing is a strategy that can be adopted to save time and money by reducing drug development timeline and research and development process cost. Hence, it is an effective alternative to conventional drug development. Different approaches for drug repurposing involve methods based on similarities in drugs, targets, or diseases. Here, we focused on the structural target similarities considering protein–protein interfaces formed by proteins involved in Ras/Raf/MEK/ERK signaling pathway. This pathway plays a role in cell signaling that regulates cell proliferation, differentiation, and apoptosis; therefore, it is highly related to cancer and tumor progression. The protein–protein interfaces studied in this work either have been predicted by PRISM according to physically interacting proteins in STRING database or obtained from Protein Data Bank. Candidates for drug repurposing are suggested considering the binding free energy prediction of the drug to the protein interface that is structurally similar to its original target by docking.

We report that HIV protease inhibitors tipranavir, indinavir, and saquinavir can bind to EGFR-ERBB3 interface. Additionally, tipranavir and indinavir can bind to EGFR-ERBB2 interface. Furthermore, we report that galantamine used in Alzheimer’s disease treatment and the antiemetic drug granisetron can bind to RAF1–BRAF interface. These protein interfaces are involved in signal transduction that activates Ras/Raf/MEK/ERK signaling pathway leading to biological processes that promote tumor growth. Hence, disruption of these interfaces may interrupt the transduction of the signals associated with cancer. Consequently, these drugs are proposed to be repurposed as anti-cancer agents.

Although our results present some candidates for drug repurposing and are important in identification of the compound to be repurposed, in-silico drug repurposing approach needs to be supported by experimental data that shows the complete effect of the drug. Thus, candidates suggested in this work should be validated experimentally and by clinical trials in future studies.

## Materials and methods

**Figure 7:**
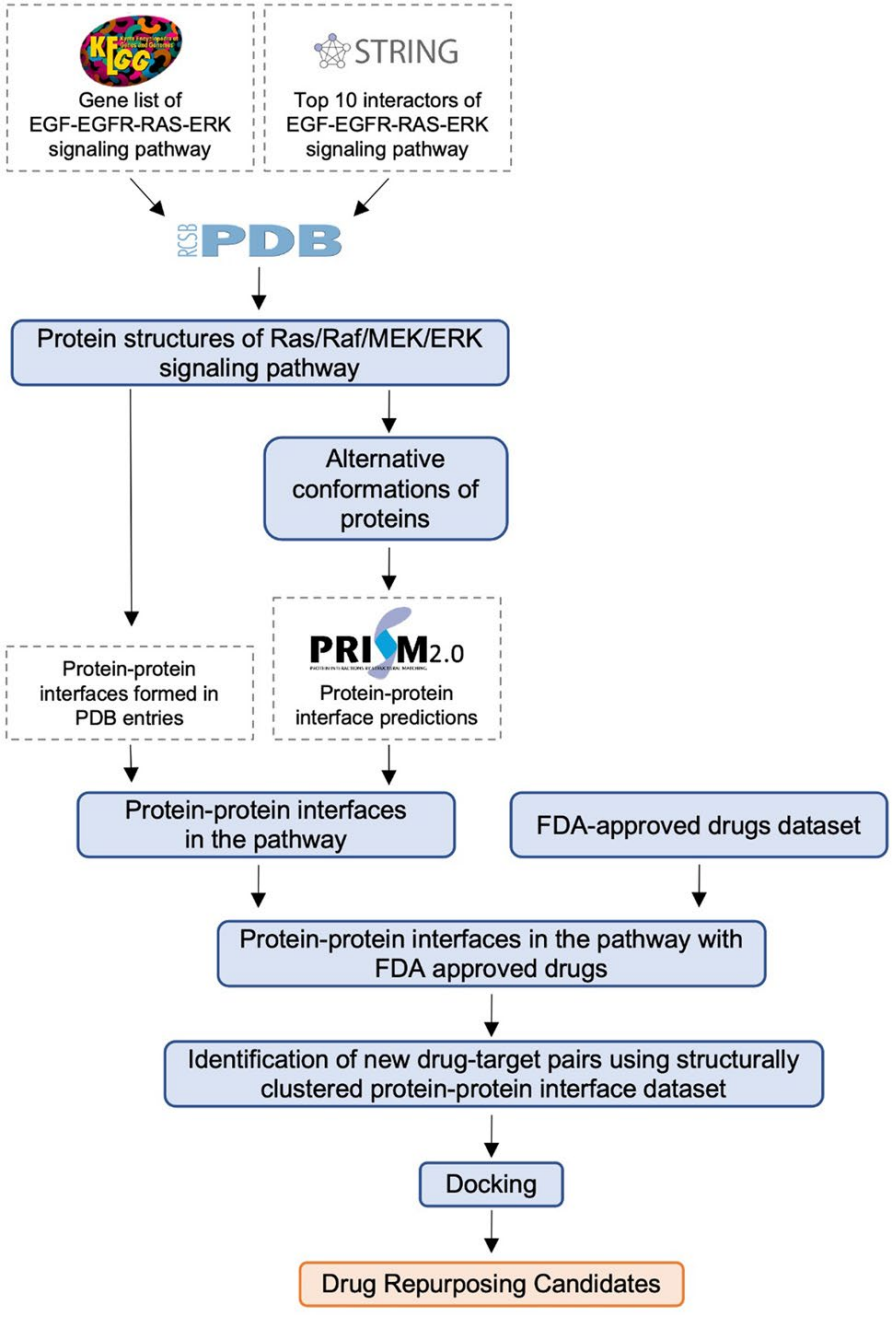
Workflow of the study. A list of proteins in the Ras/Raf/MEK/ERK signaling pathway is constructed from the KEGG and STRING databases. Their three-dimensional structures are found in the Protein Data Bank (PDB). These structures are grouped to obtain alternative conformations. The protein–protein interfaces formed by these proteins in PDB entries are determined or predicted via a template-based protein–protein docking tool. The interfaces with approved drugs are filtered using the FDA-approved drugs dataset and new drug–target pairs are identified using a structurally clustered protein–protein interface dataset. Then, docking is performed to propose drug repurposing candidates.

The basis of this study is that if a drug can bind to a protein–protein interface, it may also bind to another interface that is structurally similar to the protein–protein interface that the drug is originally bound to. Since our study focuses on Ras/Raf/MEK/ERK signaling pathway, we extracted the structures of the proteins in this pathway. Then, the alternative conformations of these proteins are determined and used in the prediction of the complexes of physically interacting proteins using PRISM^33^, a prediction tool for protein–protein interactions at the structural level, if they are not available in the literature. Following that, protein–protein interfaces with drugs are filtered to suggest new targets for these drugs using a structurally similar protein–protein interface dataset and docking. Figure 7 illustrates the workflow of this study.

### Protein structures of Ras/Raf/MEK/ERK signaling pathway

The gene list for the EGF–EGFR–RAS–ERK signaling pathway (N00001) under MAPK signaling pathway is obtained from the Kyoto Encyclopedia of Genes and Genomes (KEGG)^36–38^. KEGG identifiers for these genes are mapped to UniProt identifiers. If more than one UniProt identifier is associated with the gene, the UniProtKB/Swiss-Prot identifier (reviewed, manually annotated) is selected. Physical interactions for the 16 proteins in the EGF–EGFR–RAS–ERK signaling pathway with the highest confidence score (≥ 0.900) and their top 10 interactors (Supplementary Table S10) are imported from the STRING database^39^. The proteins in EGF–EGFR–RAS–ERK signaling pathway and their top 10 interactors form our set of pathway proteins.

Following that, PDB entries for these UniProt identifiers are found using “idmapping_selected.tab.gz” file from the UniProt website^132^. Since PDB is redundant and some PDB entries are very similar, proteins with 95% sequence identity and 2 Å RMSD value are grouped for each UniProt identifier. One representative is kept for each group. Proteins having less than 30 residues are eliminated^133^. These steps provided us with multiple conformations of the pathway proteins introducing dynamics in the predictions.

### Protein–protein interfaces in the pathway

Protein–protein interfaces used in this work are either predicted by the PRISM web server or found in PDB. PRISM predicts interactions between two proteins according to the similarity between the surfaces of target proteins and each side of a template interface. Physically interacting protein pairs according to STRING are sent to the PRISM web server as target proteins. PRISM results consist of an interface template, binding energy, protein complex structure and interface residues. PRISM may give none or multiple results for each target protein pair.

Additionally, PDB entries involving one of the proteins in the EGF–EGFR–RAS–ERK signaling pathway are found. Only the proteins listed under the EGF–EGFR–RAS–ERK signaling pathway in KEGG are included to avoid 2nd shell interactors. Protein–protein interfaces formed in these PDB entries are used in the following steps. If the distance between two atoms is less than the sum of their van der Waals radii plus a tolerance of 0.5 Å, they are considered contacting. If there are at least five contacting residues at each protein chain, they are considered to be forming an interface.

### Filtering of protein–protein interfaces with Food and Drug Administration (FDA) approved drugs

FDA-approved drugs are listed in the ZINC database^134^ and those in PDB are identified to form the FDA-approved drugs dataset used in this work (Supplementary Dataset S1)^135^. Glycerol (PDB Ligand ID:GOL) and isopropyl alcohol (PDB Ligand ID:IPA) that are present in FDA-approved drugs dataset are highly observed in PDB entries. However, these molecules are mostly used in the structure determination step as precipitant or to protect proteins when frozen^136,137^. Hence, glycerol and isopropyl alcohol are excluded from the FDA-approved drugs dataset in this step.

Protein–protein interfaces in the EGF–EGFR–RAS–ERK signaling pathway with FDA-approved drugs are filtered by mapping and combining ligands at the interface residues using data from PDBsum^138^ with the FDA-approved drug dataset (Supplementary Dataset S2)^135^. Protein–protein interfaces predicted by PRISM and interfaces from the PDB are studied separately.

### Identification of new drug–target pairs

To propose new drug target pairs, a dataset consisting of clusters of structurally similar protein–protein interfaces is used (Supplementary Dataset S3)^43^. This dataset is constructed by clustering protein interfaces in PDB entries with an Interface-Similarity score (IS-score) of 0.311 according to iAlign^139^. SparseHC^140^, a hierarchical clustering algorithm, is used in the clustering.

Two cases are considered to propose new drug–target pairs (Fig. 3):

- Repurposing To: A drug bound to one of the interfaces in a protein–protein interface cluster may bind to an interface in the same cluster, and the protein is in the Ras/Raf/MEK/ERK pathway.
- Repurposing From: A drug bound to a protein interface in Ras/Raf/MEK/ERK pathway may also bind to another protein interface that is in the same cluster, and the protein is not in the Ras/Raf/MEK/ERK pathway.

### Docking

Python package of AutoDock Vina^141^ is used in this work for docking. Additionally BioPython^142^ and NumPy packages^143^ in Python, Chimera^48^ and Open Babel^144^ are used. The 3-dimensional structures of drugs at reference pH are downloaded from ZINC database^134^. Both receptor and ligand structures are prepared for docking using codes in MGL Tools^145^. The size and the center of the docking box is adjusted to include interface residues (Supplementary Dataset S4) in the box. Docking is performed with exhaustiveness of 8 because it is the best option for the prediction of binding energy considering the increased computation time with a higher exhaustiveness^44^. Details are presented in Supplementary Text S2.

### Control set for docking

A control set of drugs are randomly selected to evaluate their energy. 35 drugs are selected from the FDA-approved drugs dataset (Supplementary Dataset S1) using random.sample() function in random module of Python. The list of drugs can be found in Supplementary Table S11. These selected drugs are docked to the interfaces that the drug repurposing candidates are reported to be binding (i.e., EGFR-ERBB2, EGFR-ERBB3 and BRAF-RAF1 interfaces). The docking procedure has been explained in the “Docking” section of “Materials and methods”.

### Cancer mutations

For the somatic mutations in cancer, missense and nonsense mutations from the COSMIC (v97) database are used^49^. Human proteins included in the proteome UP000005640^132^ are considered. Using PDBe API^112^, UniProt mappings for each PDB ID from SIFTS^111^ are obtained. For the start and end residue numbers, author residue numbers are considered. The residue names are compared, the start and end residues are manually adjusted to be consistent if they do not match. The mutations in the whole chain are listed and compared with interface residues to find the ones that are at the interface. For the mutations located at the interfaces where the drug repurposing candidates are proposed to be binding, SIFT^50^ is used to predict the functional effect of the mutation on the protein function.

### Experimental data of drug sensitivity

Drug sensitivities of cancer cell lines to selected drugs are extracted from DepMap^51^ (https://depmap.org/portal/). If the sensitivity data of a cell line is not available for all of the drugs, that cell line is omitted. Negative values suggest that the growth of treated cells is less than that of the control cells. The data on DepMap was obtained using PRISM viability assay where barcoded cell lines were exposed to the compound for five days and the abundance of mRNA barcodes was detected using Luminex MagPlex Microspheres to estimate cell viability in comparison to the control group^51^. PRISM Repurposing Primary Screen dataset is used in this study.

### Supplementary Information

Supplementary Information.

Supplementary Figures.

## Data Availability

PRISM is accessible through the PRISM webserver (https://cosbi.ku.edu.tr/prism/). The codes for grouping the alternative conformations are available on GitHub (https://github.com/ku-cosbi/ppi-network-alternative). All PRISM results and docking results of the proposed drugs are available on GitHub (https://github.com/ku-cosbi/RasPathwayDrugRepurposing). Drug sensitivity data can be accessed from DepMap portal (https://depmap.org/portal/) (Accessed 18 Aug. 2023). Other data used in this work can be found in supplementary materials.

## Abbreviations

CDK4: Cyclin-dependent kinase 4
CDK6: Cyclin-dependent kinase 6
CDKN2D: Cyclin-dependent kinase inhibitor 2D
EGF: Epidermal growth factor
EGFR: Epidermal growth factor receptor
ERK: Extracellular signal-regulated kinase
FDA: Food and Drug Administration
HIV: Human immunodeficiency virus
HTR4: Human serotonin receptor 4
MAPK: Mitogen-activated protein kinase
MEK: Mitogen-activated protein kinase kinase
mTOR: Mammalian target of rapamycin
PDB: Protein Data Bank
PI3K: Phosphatidylinositol-3 kinase
PPI: Protein–protein interaction
