# Supplementary material for: Conformational diversity and protein–protein interfaces in drug repurposing in Ras signaling pathway
Source: Sci Rep. 2024 Jan 12;14:1239. doi: 10.1038/s41598-023-50913-8 (PMC10786864; doi:10.1038/s41598-023-50913-8)
Supplement: Supplementary file 2 — Supplementary Figures. [file 41598_2023_50913_MOESM2_ESM.docx]

**Conformational diversity and protein-protein interfaces in drug repurposing in Ras signaling pathway**

Ahenk Zeynep Sayin^1^, Zeynep Abali^2^, Simge Senyuz^2^, Fatma Cankara^2^, Attila Gursoy^3^, Ozlem Keskin^1*^

^1^ Department of Chemical and Biological Engineering, Koc University, Istanbul, 34450, Turkey
^2^ Department of Computational Sciences and Engineering, Koc University, Istanbul, 34450, Turkey
^3^ Department of Computer Engineering, Koc University, Istanbul, 34450, Turkey

^*^Corresponding author:
Ozlem Keskin
College of Engineering, Chemical and Biological Engineering
Rumeli Feneri Yolu Sariyer, 34450, Istanbul, Turkey
Voice: +90-212-338-1538
Fax: +90-212-338-1548
E-mail: okeskin@ku.edu.tr


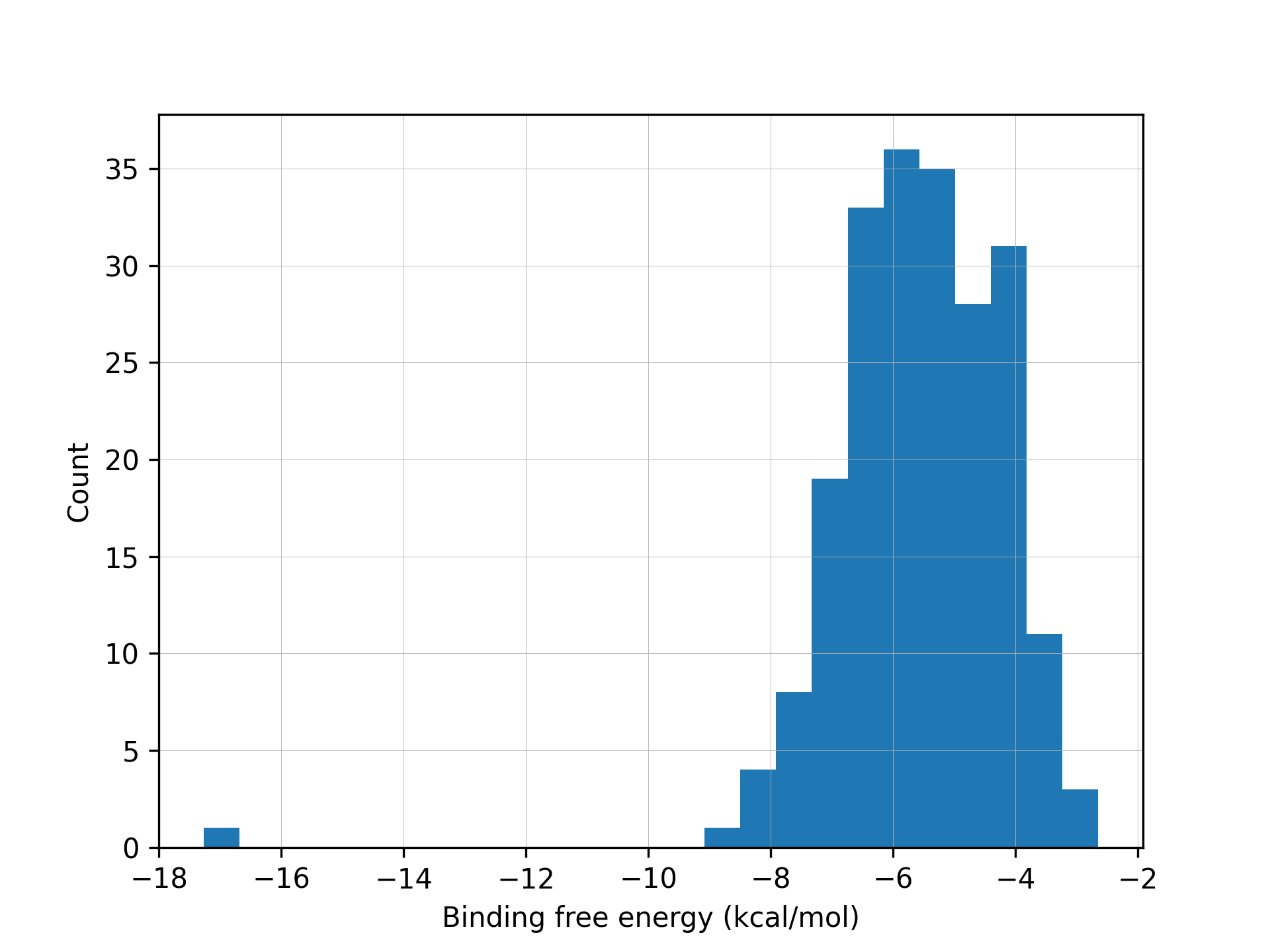


**Figure S1.** Distribution of the docking scores of randomly selected control set.


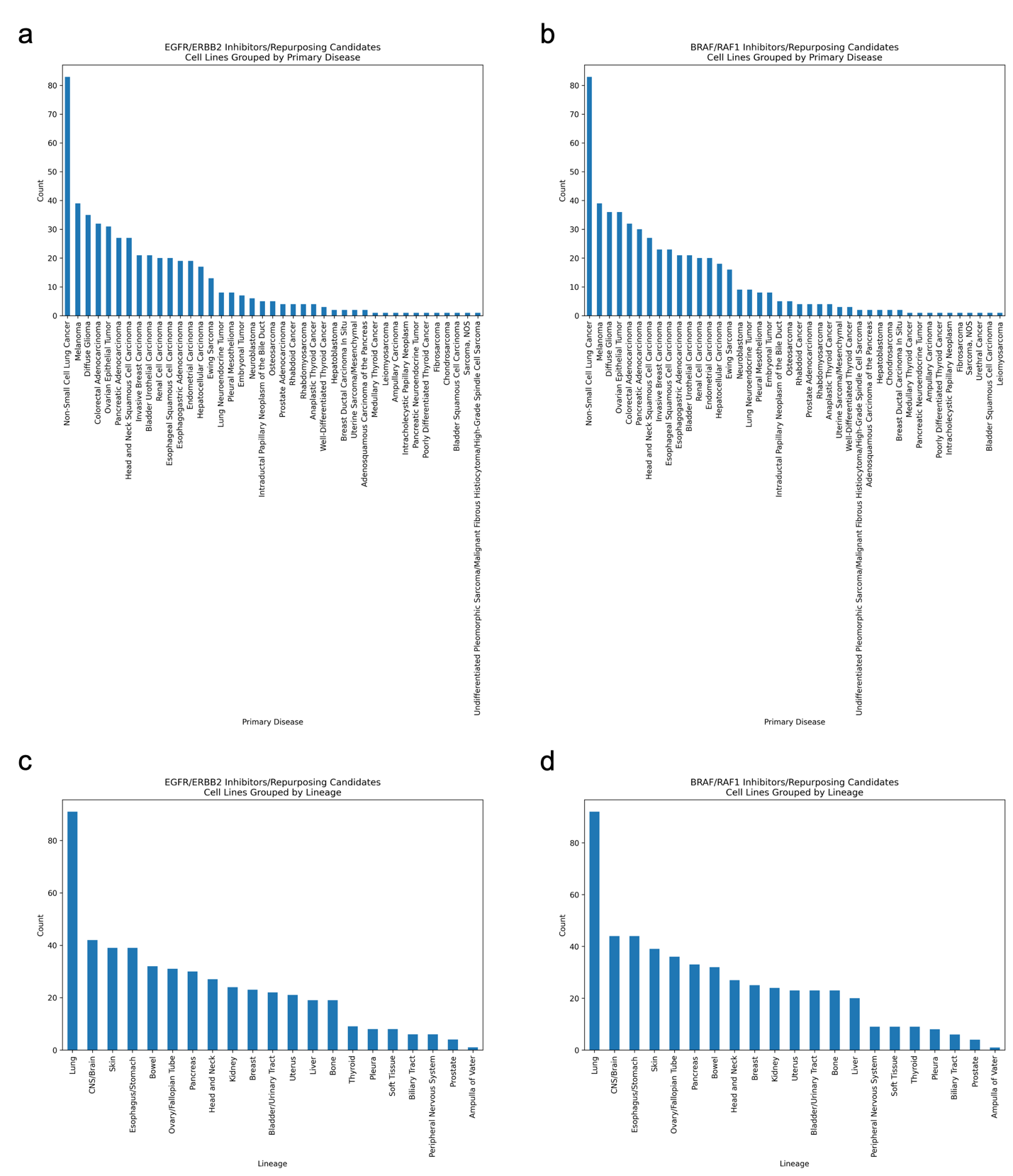


**Figure S2.** Characterization of the cell lines on DepMap used in the drug sensitivity analysis in this study. (a) Cell lines used in comparison of EGFR and/or ERBB2 inhibitors and drug repurposing candidates proposed in this study grouped by their primary diseases. (b) Cell lines used in comparison of BRAF and/or RAF1 inhibitors and drug repurposing candidates proposed in this study grouped by their primary diseases. (c) Cell lines used in comparison of EGFR and/or ERBB2 inhibitors and drug repurposing candidates proposed in this study grouped by their lineage. (d) Cell lines used in the comparison of BRAF and/or RAF1 inhibitors and drug repurposing candidates proposed in this study grouped by their lineage.
